# ERα Mediates Estrogen-Induced Expression of the Breast Cancer Metastasis Suppressor Gene *BRMS1*

**DOI:** 10.3390/ijms17020158

**Published:** 2016-01-26

**Authors:** Hongtao Ma, Lauren S. Gollahon

**Affiliations:** Department of Biological Sciences, Texas Tech University, 2901 Main St. Suite 108, Lubbock, TX 79409, USA; htma@cn.imshealth.com

**Keywords:** breast cancer, metastasis, estrogen, estrogen receptor, BRMS1

## Abstract

Recently, estrogen has been reported as putatively inhibiting cancer cell invasion and motility. This information is in direct contrast to the paradigm of estrogen as a tumor promoter. However, data suggests that the effects of estrogen are modulated by the receptor isoform with which it interacts. In order to gain a clearer understanding of the role of estrogen in potentially suppressing breast cancer metastasis, we investigated the regulation of estrogen and its receptor on the downstream target gene, breast cancer metastasis suppressor 1 (*BRMS1*) in MCF-7, SKBR3, TTU-1 and MDA-MB-231 breast cancer cells. Our results showed that estrogen increased the transcription and expression of BRMS1 in the ERα positive breast cancer cell line, MCF-7. Additionally, the ERα specific agonist PPT also induced the transcription and expression of BRMS1. However, the two remaining estrogen receptor (ER) subtype agonists had no effect on BRMS1 expression. In order to further examine the influence of ERα on BRMS1 expression, ERα expression was knocked down using siRNA (siERα). Western blot analysis showed that siERα reduced estrogen-induced and PPT-induced BRMS1 expression. In summary, this study demonstrates estrogen, via its α receptor, positively regulates the expression of BRMS1, providing new insight into a potential inhibitory effect of estrogen on metastasis suppression.

## 1. Introduction

Breast cancer is the leading female cancer in the United States and the leading cause of cancer-associated death in women. One of the risk factors associated with breast cancer development is a woman’s lifetime exposure to estrogen [1,2]. Estrogen performs its function through its estrogen receptor (ER) in target cells [3]. However, prior (and current) studies gauge the effect of estrogen on the ER in general; not delineated based on the different subtypes. Evidence is slowly emerging that demonstrate distinctions in the functions of ER subtypes with respect to cancer [4]. Our study looked specifically at the effect of the estrogen on the known ER subtypes to determine if there was a correlation with activation of a putative down-stream target involved in metastasis.

Two types of estrogen receptors exist. The nuclear estrogen receptor (nER) is a member of the nuclear hormone family of intracellular receptors. nERs exist as two isoforms, α and β. The estrogen G protein coupled receptor GPR30 is a cytoplasmic receptor, which is predominantly associated with the endoplasmic reticulum [5,6]. Upon estrogen binding, several sites along the nER undergo phosphorylation and subsequent conformational changes freeing the receptor from the Hsp90 aggregate. At this point, the activated receptor subunits dimerize and bind to the estrogen response element (ERE) in the promoter regions of target genes in the nucleus [7,8,9]. In addition, the nER may also regulate gene expression by binding to transcriptional complexes on other promoter sites, e.g., Sp1 or AP1 [10,11,12,13,14,15]. In contrast, upon estrogen binding, the membrane-bound estrogen receptor GPR30 activates G proteins, which in turn activate adenyl cyclases, Src, and SphK. These kinases mediate subsequent transcriptional responses [16,17,18].

It is well known that estrogen promotes the proliferation of ER positive cancer cells both *in vivo* and *in vitro* [19,20,21,22,23,24,25]. In contrast, other studies have reported estrogen-induced inhibition of cancer cell invasion and motility [23,26,27,28]. However, the mechanism of this inhibitory effect remains unknown. Recent data suggest that the ER may mediate transcription of metastasis-associated genes, e.g., nm23, KAI1, KiSS1, and MTA [29,30,31,32,33]. Breast cancer metastasis suppressor 1 (BRMS1), a known metastasis suppressor gene, has been shown to suppress metastasis of a variety of tumor cell lines, including breast, melanoma, bladder, ovarian and lung [34,35,36,37,38]. Several studies have indicated a possible link between the ER and BRMS1. One study, involving 161 breast carcinoma specimens, showed that the level of BRMS1 mRNA expression was marginally higher in ERα-positive group than in the ERα-negative group [39]. An analysis of 238 tissue samples by microarray showed a trend for BRMS1 towards a positive association with the ER [40]. Frolova *et al.* [41] found that nuclear expression of BRMS1 was positively correlated with expression of ER. ER-negative breast cancers were significantly more likely than ER-positive cancers to have low BRMS1, and loss of nuclear BRMS1 was associated with ER-negative cancers. Based on this information, we investigated whether BRMS1 expression in breast cancer cells was mediated through a specific estrogen receptor in response to estrogen. Our results demonstrate that BRMS1 expression is upregulated by estrogen stimulation through its ERα receptor.

## 2. Results

### 2.1. ER Subtype Expression in MCF-7, TTU-1, MDA-MB-231, and SKBR3 Cell Lines

**Figure 1:**
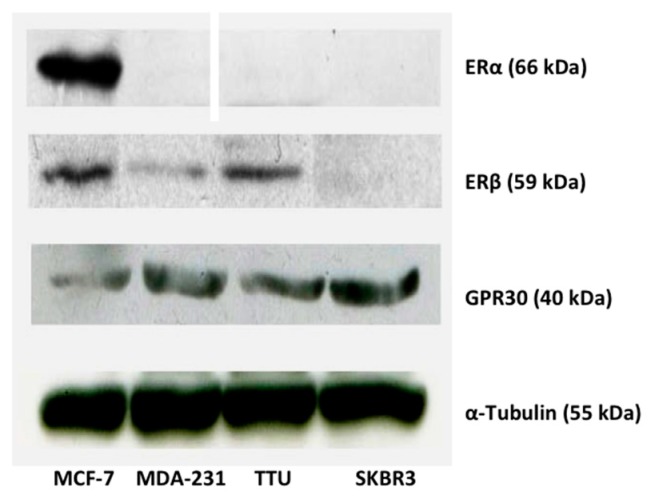
Protein expression levels for breast cancer cells demonstrating different ER subtypes. Western analysis was performed against ERα, ERβ, GPR30 on MCF-7, MDA-MB-231, TTU-1, and SKBR3 cells using 40 µg of total cell lysate. α-tubulin was used as a loading control. Upon detection, protein was quantitated using Image J.

In order to test the effects of ER influence on BRMS1, the ER subtype expression in MCF-7, TTU-1, MDA-MB-231, and SKBR3 cell lines was investigated by Western analysis. Results showed that the expression of the ER subtype was dependent upon the characteristics of the individual cancer cell line (Figure 1). MCF-7 cells, (histopathologically ER+) expressed all three ER subtypes, ERα, ERβ, and GPR30. Neither TTU-1 (histopathologically ER−) nor MDA-MB-231 (histopathologically ER−) cells expressed ERα. SKBR3 cells (histopathologically ER−) only expressed GPR30 (Figure 1). Based on protein expression levels, MCF-7, TTU-1 and SKBR3 were further analyzed.

### 2.2. E_2_ and the ERα Agonist PPT Induced BRMS1 Expression in MCF-7 Cells

Since DMSO was used as the vehicle to dissolve E_2_, PPT, DPN and G-1 (for full name details, see abbreviations list) a control experiment was performed with cells treated at the same DMSO concentration used for chemical dissolution, over the same time points. Results show insignificant effects of DMSO *versus* untreated cells (Figure S1). Additionally, these control experiments demonstrated no significant changes in BRMS1 expression levels in cells treated with DMSO for all time points (Figure S1). Therefore, DMSO 2 h controls were used to demonstrate background BRMS1 levels throughout the remainder of the experiment.

**Figure 2:**
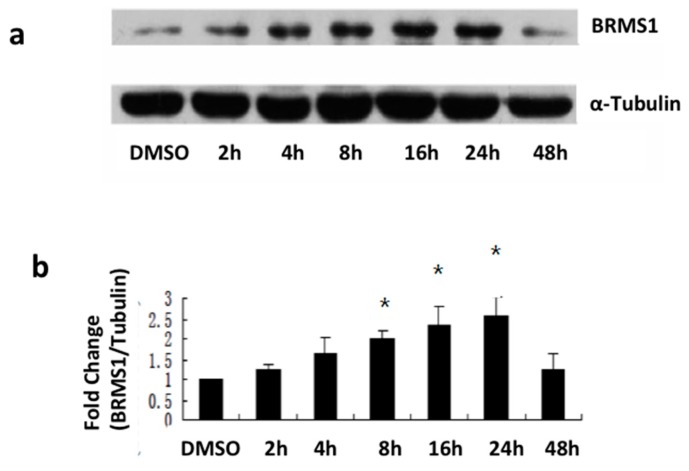
Western analysis of BRMS1 protein expression induced by E_2_ in MCF-7 cells. (**a**) Cells were incubated with culture media containing 10 nM E_2_ for 2, 4, 8, 16, 24, and 48 h. Control cells were incubated with culture media containing 10 nM DMSO for 2 h. At each time interval, cells were harvested and 20 μg of the total protein was loaded for each sample to determine BRMS1 expression by Western blot. α-tubulin was used as the loading control. Each band present was quantified using Image J and a set area encompassing the BRMS1 band was divided by the same area for α-tubulin from the same lane to determine the relative amount of BRMS1 expression. The DMSO value was set to 1 for comparisons; (**b**) The bar graph summarizes BRMS1 protein normalized to α-tubulin from the same lane in three independent experiments. Error bars were the average of three independent experiments ± SD. * indicates statistical significance at the *p* < 0.05 level compared to DMSO.

**Figure 3:**
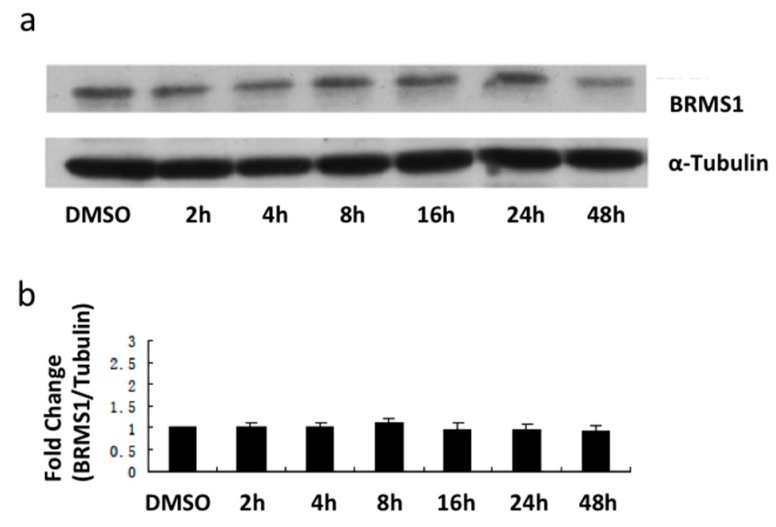

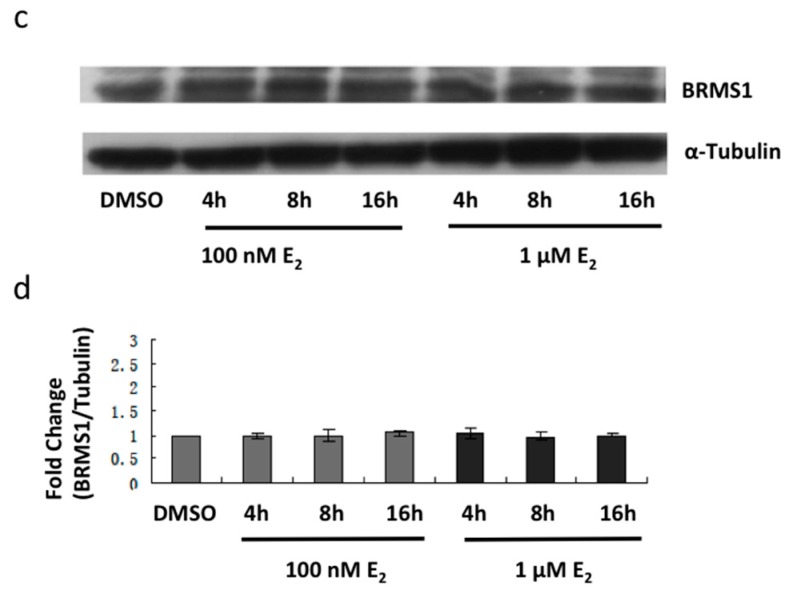
Analysis of BRMS1 protein expression induced by E_2_ in SKBR3 cells. (**a**) SKBR3 cells were incubated with culture media containing 10 nM E_2_ for 2, 4, 8, 16, 24, and 48 h. Control cells were incubated with culture media containing 10 nM DMSO; (**b**) Bar graph summarizes BRMS1 protein normalized to α -tubulin from the same lane from three independent experiments from (**a**); (**c**) SKBR3 cells were incubated with culture media containing 100 nM E_2_ or 1 μM E_2_ for 4, 8, and 16 h. Control cells were incubated with culture media containing 100 nM DMSO for 2 h; (**d**) Bar graph summarizes BRMS1 protein normalized to α-tubulin from the same lane from three independent experiments from (**c**). At each time interval, cells were harvested and 20 μg of the total protein was loaded for each sample to determine BRMS1 expression by Western blot. For all samples, α-tubulin was used as the loading control. Each band present was quantified using Image J and graphed as previously described. The bar graphs and error bars represent the average of three independent experiments ± S.D. No statistical significance was observed.

To determine whether BRMS1 expression was modulated in response to E_2_, cells were incubated with culture media containing 10 nM E_2_, the endogenous estrogen receptor agonist (*Kd* = 0.1 nM and 0.4 nM for ERα and ERβ, respectively [42]) for 48 h. It is also a high affinity ligand for the estrogen membrane receptor GPR30 (*Kd* = 9.0 nM) [43]. Western analysis demonstrated that 10 nM E_2_ increased BRMS1 expression prior to 24 h in MCF-7 cells (Figure 2a). Compared to DMSO, the expression of BRMS1 was significantly increased at 8, 16, and 24 h (*p* ≤ 0.05) (Figure 2b). However, BRMS1 expression did not significantly change in SKBR3 (Figure 3) or TTU-1 (Figure S2) cells in response to E_2_ (10 nM–1 μM) for up to 48 h. These results suggested that E_2_ positively regulates BRMS1 expression via the ERα.

**Figure 4:**
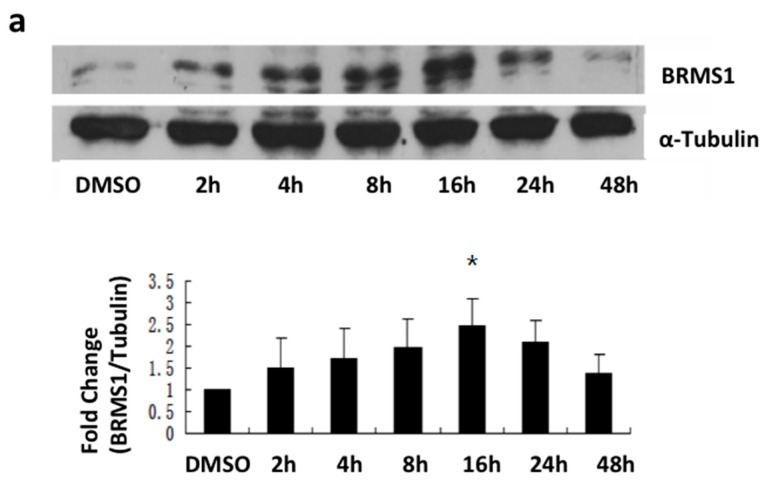

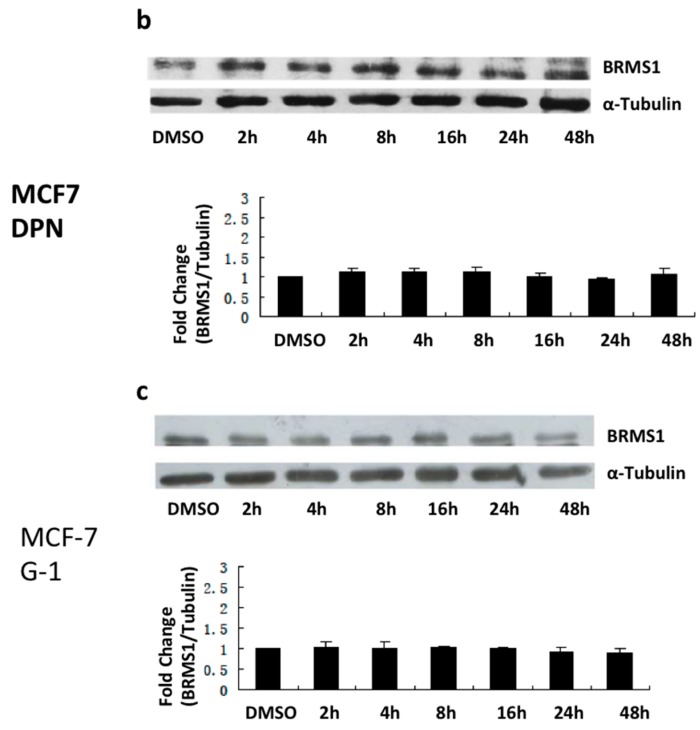
Western blot results of PPT, DPN and G-1 effects on induction of BRMS1 protein expression in MCF-7 cells. MCF7 cells were incubated with culture media containing (**a**) 10 nM PPT; (**b**) 10 nM DPN or (**c**) 100 nM G-1 for 2, 4, 8, 16, 24 and 48 h. Control cells were incubated with culture media containing 10 nM DMSO (**a**, **b**) or 100 nM (**c**) for 2 h. Proteins were quantitated as previously described. Graphs and error bars represent the average of three independent experiments ± SD. * indicates statistical significance at the *p* < 0.05 level compared to DMSO.

To further validate this observation, cells were exposed to ER agonists to activate specific ER subtypes. PPT is an ERα selective agonist, whereas DPN is an ERβ selective agonist [44]. G-1 is a known agonist for GPR30 [16]. Changes in BRMS1 expression were determined by immunoblot. Results showed that 10 nM PPT increased BRMS1 expression for up to 24 h in MCF-7 (Figure 4a). Compared with DMSO controls, the expression of BRMS1 was significantly increased at 16 h (*p* ≤ 0.05) (Figure 4a). However, the ERβ and GPR30 agonists DPN and G-1, respectively, did not change BRMS1 expression in these ER subtype positive cells for up to 48 h. Incubation of MCF-7 cells with DPN and G-1 at concentrations ranging from 10 nM to 1 μM reflected similar results (data not shown). These results confirmed our initial data indicating that ERα activation induced BRMS1 expression.

### 2.3. E_2_ and the ERα Agonist PPT Increased BRMS1 Transcription in MCF-7 Cells

**Figure 5:**
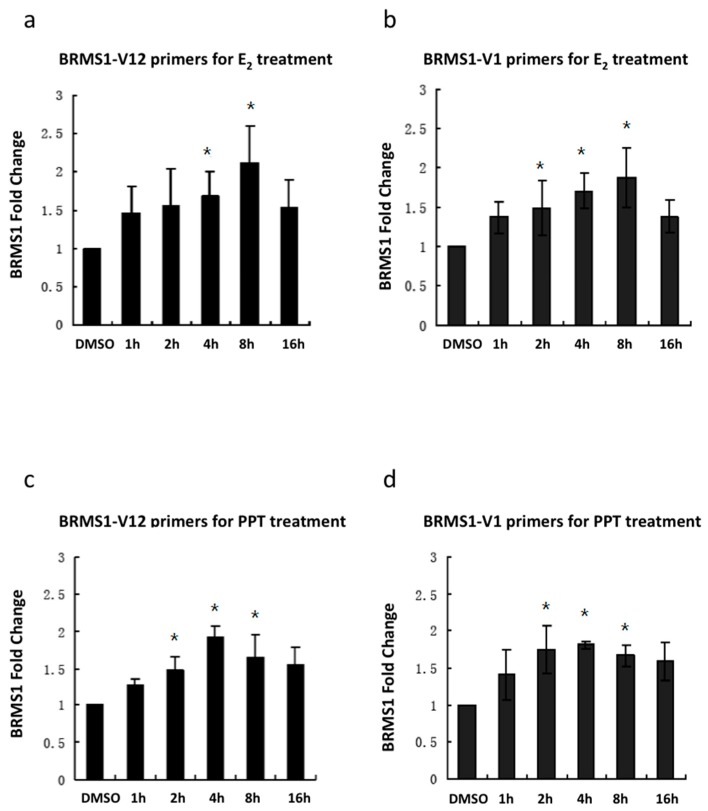
BRMS1 transcription was induced by treatment with E_2_ or PPT. BRMS1 transcription for BRMS1 V12 primers (**a**) and BRMS1 V1 primers (**b**) after treatment with 10 nM E_2_ demonstrated peak induction at 8 h; BRMS1 transcription for BRMS1 V12 primers (**c**) and BRMS1 V1 primers (**d**) after treatment with 10 nM PPT showed much earlier peak induction between 2 and 4 h. Values with error bars are the average of three independent experiments ± SD. * indicates statistical significance at the *p* < 0.05, compared to DMSO.

The next question investigated was whether the results observed at the protein level were also evident at the RNA level. To address this, real-time quantitative PCR (RT-PCR) was used to analyze the effect of E_2_ and PPT on BRMS1 transcription. Since there are multiple splice variants of BRMS1 listed in the NCBI database, we chose to analyze the BRMS1 transcript NM_015399, which was first reported and encodes the most abundant transcript [34]. Additionally, the BRMS1 transcript NM_001024957 uses an alternative splice site in exon 10. Thus, two pairs of BRMS1 primers, BRMS1-V1 and BRMS1-V12 were analyzed for transcriptional activation of BRMS1 in MCF-7 cells following incubation with culture media containing 10 nM E_2_ and PPT. Optimization experiments included an initial time course of 1, 2, 4, 8 and 16 h (Figure 5). Results showed that 10 nM E_2_ increased BRMS1 expression up to 16 h (Figure 5a,b). At 8 h, the mRNA level of BRMS1 was significantly increased for both pairs of primers. There was also a significant increase of BRMS1 transcription at 2 h for BRMS1-V1 primers. In contrast, we observed attenuation in the time to significantly increased mRNA levels of BRMS1 to 4 and 8 h for the BRMS1-V12 primers (Figure 5a). Furthermore, the highest transcription peak appeared at 8 h for both pairs of primers (Figure 5a,b). Moreover, RT-PCR results showed that 10 nM PPT increased BRMS1 expression up to 16 h as well. At 2, 4, and 8 h, the mRNA level of BRMS1 was significantly increased for both sets of primers (Figure 5c,d) with the highest transcription peak occurring at 4 h for both primers (Figure 5c,d).

### 2.4. siRNA Inhibition of ERα Expression Decreased E_2_ and PPT-Induced BRMS1 Expression

**Figure 6:**
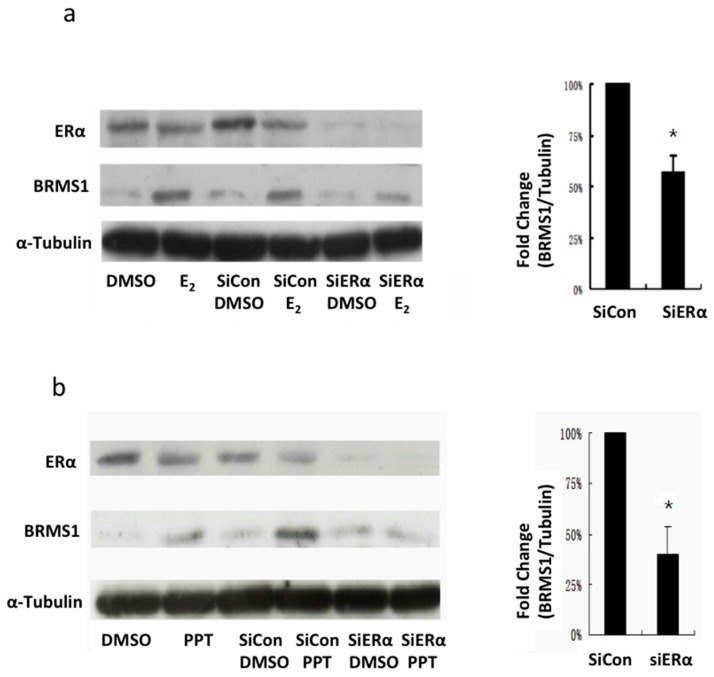
The effects of siERα on BRMS1 expression in MCF-7 cells after treatment with E_2_ or PPT. MCF-7 cells were transfected with siERα for 48 h and then treated with either E_2_, PPT, or DMSO for 16 h. siControl cells were incubated in the same volume of transfection reagent without siERα. Each lane was loaded with 20 μg and membranes were probed with monoclonal antibodies against ERα, BRMS1, or α-tubulin as loading control. Controls are untransfected cells treated with DMSO, PPT or E_2_. (**a**) Western blot demonstrating knockdown of ERα and corresponding BRMS1 protein expression from MCF-7 cells treated with 10 nM E_2_. The bar graph (**right panel**) summarizes BRMS1 protein normalized to α-tubulin from control and SiERα from the same lane in three independent experiments; (**b**) Western blot demonstrating knockdown of ERα and corresponding BRMS1 protein expression from MCF-7 cells treated with 10 nM PPT. The bar graph (**right panel**) summarizes BRMS1 protein normalized to α-tubulin from control and SiERα from the same lane in three independent experiments. Values with error bars were the average of three independent experiments ± SD. * indicates statistical significance at the *p* < 0.05, compared to siControl for E_2_ or PPT treatment.

To investigate BRMS1 expression in response to E_2_ and PPT-induced activation following ERα knockdown, MCF-7 cells were transfected with 100 nM siERα for 48 h followed by treatment with 10 nM E_2_ for 24 h or 10 nM PPT for 16 h. Results are shown in Figure 6. DMSO was used as control. Control cells were incubated in the same volume of transfection reagent without siERα and treated with E_2_ or PPT following the same conditions described above. Western analysis showed that DMSO and SiRNA control with DMSO did not affect ERα expression (Figure 6a,b). Negative control experiments were also performed using siRNA-A (Santa Cruz, Santa Cruz, LA, USA) to demonstrate specificity of siERα target effects (Figure S3). In the presence of E_2_ and PPT, ERα expression remained unchanged, but BRMS1 expression increased as previously observed. With the addition of siERα knockdown, both E_2_-induced BRMS1 expression and PPT-induced BRMS1 expression was significantly decreased by ~40% and 60%, respectively (*p* ≤ 0.05) (Figure 6a,b). Taken together, these results demonstrate that ERα contributed to the changes in BRMS1 expression induced by E_2_.

## 3. Discussion

In this study, we investigated whether expression of the breast cancer metastasis suppressor gene BRMS1 changed in response to estrogen exposure. Additionally, we also addressed the question of whether a specific ER subtype was responsible for modulating BRMS1 expression. Here, we report for the first time, that BRMS1 is a downstream target in ER positive breast cancer cells and that its expression is modulated by the ERα in the presence of estrogen or an ERα agonist. Our results suggest that estrogen can have a dual effect on the ERα. On one hand, it is known to stimulate tumor cell growth [45,46,47]. However, in this study, we demonstrate that it may also inhibit tumor cell invasion by upregulation of the breast cancer metastasis suppressor gene BRMS1.

By treating breast cancer cells with differential expression of the ER subtypes with E_2_, we observed that the regulation of E_2_ on the metastasis suppressor gene BRMS1 induced a time-dependent increase in the amount of BRMS1 protein produced only in ERα positive MCF-7 cells. This suggested that E_2_, via its α receptor, positively regulated the expression of BRMS1. In order to confirm this finding, three ER subtypes were activated using receptor specific agonists to determine whether BRMS1 expression was changed. Again, the expression of BRMS1 only showed a time-dependent increase in response to the ERα agonist PPT. In contrast, agonists for ERβ (DPN) and GPR30 (G-1) did not change BRMS1 expression in any of the cell lines tested. The effect of this E_2_-ERα-BRMS1 relationship was further shown to occur at the transcriptional level. RT PCR showed a corresponding increase in BRMS1 mRNA levels with either E_2_ or PPT treatment. However, this event occurred prior to BRMS1 protein expression, where mRNA increased from 1 to 8 h, but protein levels continued to increase until 16 h (PPT) or 24 h (E_2_). In eukaryotic cells, time-delayed gene expression is essential for the function of the circadian clock, somite-segmentation clock, and mitosis [48]. Time-delay is mainly associated with the regulation of protein stability by phosphorylation and ubiquitination [49]. The mechanisms involved in this process include proteasome-dependent proteolysis and negative feedback regulation [50]. Interestingly, others have reported a similar estrogen-triggered delay in gene expression as well [51,52]. Recent studies have shown that circadian clock gene expression is maintained in ER+, low metastatic breast cancers, whereas, the loss of circadian clock gene expression is associated with more aggressive breast cancers [53]. More study is warranted on the importance of this phenomenon with respect to BRMS1 activation.

**Figure 7:**
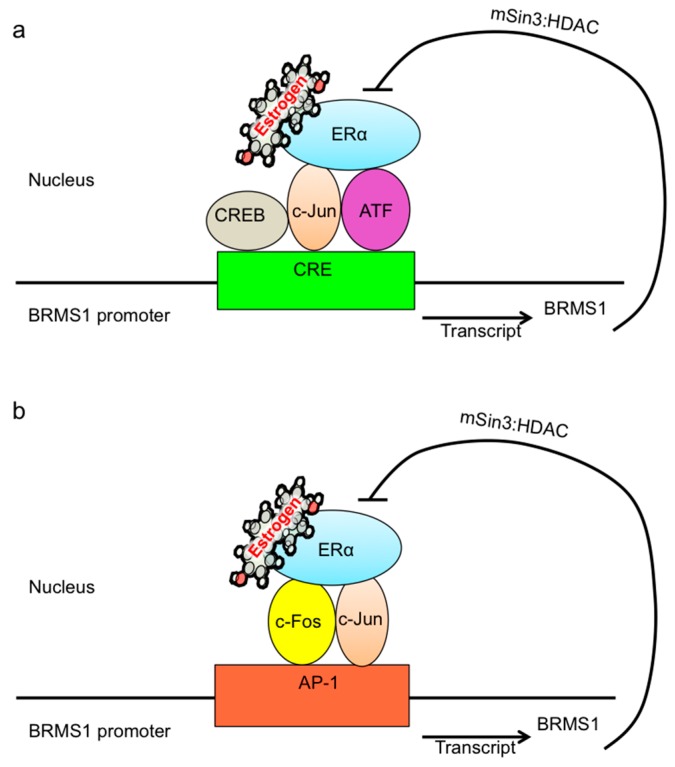
A proposed model for the relationship between ERα and BRMS1. (**a**) The BRMS1 5′ upstream region shows several putative regulatory elements, one of them is the binding site for CREB. ERα may regulate gene expression as a co-regulator on other promoter sites. ERα has been shown to bind c-Jun/ATF proteins, which bind to the CRE of the cyclin D1 promoter, inducing its expression. This suggests that ERα may induce BRMS1 expression by binding transcriptional complexes on the CRE in the promoter region of BRMS1; (**b**) It has been shown that ERα upregulates gene transcription by binding the AP-1 complex on AP-1 response elements. This information, taken together, suggests that ERα may induce BRMS1 expression through binding transcriptional complexes as a co-activator on other promoter sites (e.g., CRE and AP-1).

To further elucidate the influence of the ERα on the expression of BRMS1, ERα was knocked down by siRNA in MCF-7 cells. Western analysis showed that siERα reduced E_2_-induced and PPT-induced BRMS1 expression by approximately ~40% and 60%, respectively. These results confirmed the observation that ERα was responsible for the E_2_-induced BRMS1 expression in MCF-7 cells (summarized in Figure 7). What are the potential consequences of these results? Studies have shown that ERα regulates target gene transcription as a transcriptional factor or co-regulator [7,9,10,14]. As a transcriptional factor, ERα binds to the ERE in the promoter region of the target gene [7,9]. To date, the ERE in the BRMS1 promoter has not been identified. Thus, ERα regulation on BRMS1 expression may not be as a transcriptional factor. However, the BRMS1 5′ upstream region shows several putative regulatory elements, and one of them is the binding site for the cAMP response element (CRE)-binding protein (CREB) [36]. As a co-regulator, ERα may regulate gene expression by binding to transcriptional complexes on other promoter sites [10]. ERα has been shown to bind c-Jun/ATF proteins, which bind to the CRE of the cyclin D1 promoter, inducing its expression [15]. This suggests that ERα may induce BRMS1 expression by binding transcriptional complexes on the CRE in the promoter region of BRMS1. In addition, the UCSC Genome Browser has predicted a putative AP-1 binding site in the BRMS1 promoter. It has been shown that ERα upregulates gene transcription by binding the AP-1 complex on AP-1 response elements [11,12]. This information, taken together, suggests that ERα may induce BRMS1 expression through binding transcriptional complexes as a co-activator on other promoter sites (e.g., CRE and AP-1) instead of the ERE.

Alternatively, BRMS1 may regulate ERα expression. Justification for this rationale is based on reports that ERα transcription was shown to be activated in human breast cancer cells by HDAC inhibition [54]. HDAC1 interacts with ERα *in vitro* and *in vivo*, and suppresses ERα transcription activity. This interaction was mediated by the AF-2 and DBD domains of ERα [55]. However, BRMS1 may interact with the mSin3:HDAC complex, including HDAC1 and HDAC2, reducing target gene transcription [56] For review of metastatic suppression of BRMS1 and Sin3 complexes, please see [57]. Lui *et al.* [58] reported that BRMS1 suppressed RelA/p65 transcriptional activity by promoting binding of HDAC1 to RelA/p65, where it deacetylates lysine K310 on RelA/p65. BRMS1 also negatively regulates uPA gene expression through recruitment of HDAC1 to the NF-κB binding site of the uPA promoter [59]. These findings indicate that BRMS1 could participate in HDAC inhibition on ERα expression. We have presented a putative model for the relationship(s) between ERα and BRMS1 in Figure 7.

There is still little known about the function of BRMS1, binding partners or its importance in suppressing metastasis. Recently, Hurst and colleagues have elucidated several important aspects of BRMS1 expression and its involvement in cellular adhesion towards suppressing metastasis [57,60,61]. Given the findings of our study, as well as previous studies discussed above, an important question that needs further investigation is whether the inhibitory effect of estrogen on developing metastases is countered by its mitogenic effect. In other words, does estrogen promote proliferation at a greater rate than it inhibits metastasis? The study of ER subtypes and their effects is gaining momentum and recent studies address this issue [53]. Moreover, another emergent area involves the role of miRNA regulation of ER subtypes and implications towards metastasis [62], which may translate to a new strategy for assessing diagnosis, treatment and prognosis of patients with ER-positive breast cancer and its association with metastasis.

## 4. Experimental Section

### 4.1. Cell Culture and Treatment

MCF-7, SKBR3 and MDA-MB-231 breast cancer cells were purchased from ATCC (Manassas, VA, USA). TTU-1 is a primary, invasive ductal cancer cell line derived in our lab [63]. These cell lines were chosen for analysis based on differences in ER expression profiles. MCF-7 and TTU-1 cells were cultured in Dulbecco’s Modified Eagle’s Medium. SKBR3 cells were maintained in phenol red free McCoy’s 5A Medium (Modified). MDA-MB-231 cells were grown in Leibovitz’s L-15 medium. All media were phenol red-free and supplemented with 10% fetal bovine serum and 50 μg/mL gentamycin sulfate. Cells were treated with 10 nM–1 μM E_2_, PPT, DPN, and G-1 (see chemical reagents below) over 48 h. DMSO was used as the vehicle control at the same concentrations and collected at the same time points to delineate vehicle effects from treatment effects on BRMS1 expression. Control results are in Supplementary Materials (Figure S1).

### 4.2. Chemical Reagents and Antibodies

ERα-selective agonist (propyl-pyrazole-triol (PPT)), ERβ-selective agonist (diarylpropionitrile (DPN)), and the GPR30 agonist were purchased from Tocris (Bristol, UK), (referred to as G-1) (±)-1-[(3aR*,4S*,9bS*)-4-(6-Bromo-1,3-benzodioxol-5-yl)-3a,4,5,9b-tetrahydro-3H-cyclopenta[c]quinolin-8-yl]- ethanone. Estradiol (E_2_) and dimethyl sulfoxide (DMSO) were purchased from Sigma-Aldrich (St. Louis, MO, USA). Antibodies were purchased as follows: mouse monoclonal anti-human ERα and anti-human ERβ from GeneTex Inc. (Irvine, CA, USA); rabbit polyclonal anti-human GPR30 from Santa Cruz; mouse monoclonal anti-human BRMS1 from Sigma-Aldrich and Santa Cruz; mouse monoclonal anti-human α-tubulin from Abcam (Cambridge, MA, USA).

### 4.3. Western Blot Analysis

Total protein (20–40 μg/lane) was separated on an 8% or 10% polyacrylamide SDS gel and transferred to a nitrocellulose membrane (Applied Biosystems, Waltham, MA, USA). The membranes were incubated with primary antibodies overnight at 4 °C at the following dilutions: ERα 1:1000; ERβ 1:1000; GPR30 1:200; BRMS1 1:300 (Santa Cruz) and 1:1000 (Sigma); α-tubulin 1:10,000. After washing, the membranes were incubated with goat anti-mouse HRP-conjugated secondary antibody (1:5000) or goat anti-rabbit HRP-conjugated secondary antibody (1:5000), for 1 h at room temperature. A chemiluminescent substrate kit (Pierce, Waltham, MA, USA) was used for signal detection on X-ray film. Each band present was quantified using Image J [64], (National Institutes of Health, Bethesda, MD, USA). A set area encompassing the BRMS1 band was divided by the same area for α-tubulin from the same lane to determine the relative amount of BRMS1 expression. The DMSO value was set to 1 for comparison.

### 4.4. RNA Isolation and Quantitative RT-PCR

Total RNA was extracted from cells using the RNeasy Mini Kit (Qiagen, Valencia, CA, USA). Five micrograms of RNA was reverse transcribed to cDNA using a cDNA synthesis kit (Invitrogen, Waltham, MA, USA) according to the manufacturer’s instructions. Reverse transcription products were then amplified using a SYBR Green Master Mix (Applied Biosystems) for real-time PCR. BRMS1 (NM_015399) was first reported and encodes the most abundant transcript [34]. BRMS1 (NM_001024957) uses an alternative splice site in exon 10. Using the SciTool software program, two pairs of BRMS1 primers were designed based on its transcripts, variant 1 and variant 2. The sequence of variant 2 is completely covered by variant 1. One pair of primers (BRMS1 V1) was suitable only to variant 1, whereas the other pair of primers (BRMS1 V12) was suitable to both variant 1 and 2. The primer pair was selected for a Tm at approximately 60 °C, amplicon of less than 150 bp, and low primer penalty. Primers sequences compared against the human genome databank using a “Blast” search revealed that all of these three pair primers were unique for *Homo sapiens* BRMS1 or GAPDH

BRMS1-V1 and BRMS1-V12 primer sequences were as follows:

BRMS1-V12: Forward-5′-ATTCAGGTGGCAGGGATCTACAAGG-3′
BRMS1-V12: Reverse-5′-GCAGCAGCTTCTCACTCTCCA-3′
BRMS1-V1: Forward-5′-CGACATCCTGGAGGACTGGACAGCCA-3′
BRMS1-V1: Reverse-5′-TGAACAGCAGGGTCAAGGTCCATCCGA-3′

The GAPDH primers used for loading control were:

Forward-5′-CACCACCATGGAGAAGGCTG-3′
Reverse-5′-GAGGCATTGCTGATGATCTTGAGG-3′

The BRMS1 and GAPDH primers were diluted to a final concentration of 10 μM with RNase-free H_2_O before use. The 2X SYBR Green master mix (containing HotStart DNA Taq polymerase, ultrapure nucleotides, real-time PCR buffer, SYBR Green dye, and the passive reference dye ROX) (Applied Biosystems), forward and reverse primers, cDNA template, and H_2_O were added based on the number of reactions (each 20 μL reaction: 10 μL SYBR Green Mix (2×), 1 μL cDNA (20 ng/1 μL), 2 μL primer pair mix (10 μM each primer), and 7 μL RNase-free H_2_O) and mixed well. Twenty microliters of reaction solution was added to each well of a 96 well plate. Each reaction included three replicates. Three reactions without reverse transcriptase and three reactions without cDNA templates in each pair of primers were tested for genomic DNA contamination and general contamination. The real time *C*t values should be greater than 35 for these negative controls. The Applied Biosystems 7500 real time PCR instrument (Biotech Core Facility, TTU, Lubbock, TX, USA) was used to run the PCR reaction plate. The two-step thermal cycling program was performed as follows:

Real time PCR program:

Step 1: 10 min at 95 °C (enzyme activation)
Step 2: 40 cycles each of 15 s at 95 °C (denature) and 1 min at 60 °C (anneal/extend).

The PCR program was followed by a dissociation program:

15 s at 95 °C, 1 min at 60 °C, and 15 s at 95 °C.

Fold change was calculated from the ΔΔ*C*t values and data were presented as relative expression to DMSO treated control cells. In order to determine BRMS1 transcription levels, the ΔΔ*C*t method was used.

1. Change all *C*t values, which were reported as greater than 35 or N/A (undetermined) to 35. Any *C*t value equal to 35 was considered a negative call.
2. Calculate the average *C*t value of each time interval treatment across three replicates.
3. Calculate the Δ*C*t for each time interval treatment in that group
Δ*C*t = average *C*t − average of GAPDH (housekeeping gene) for that group (1)
4. Calculate ΔΔ*C*t
ΔΔ*C*t = Δ*C*t − Δ*C*t control (2)
5. Calculate the fold-change
2^−ΔΔ*C*t^ = 2^−Δ*C*t experiment^/2^−Δ*C*t control^ (3)

If the fold-change is greater than 1, the result is considered as a fold upregulation. If the fold-change is less than 1, the result is reported as a fold down-regulation.

### 4.5. siRNA Knockdown

Twenty-four hours before transfection, 2 × 10^5^ cells were plated into each well of a 6-well plate containing antibiotic-free medium. Using Lipofectamine™ 2000 (Invitrogen), a 25 bp siRNA against ERα (siERα) (Invitrogen) was transfected into MCF-7 cells, at a concentration of 100 nM, according to the manufacturer’s protocol. Control cells were incubated in the same volume of transfection reagents without siERα. Forty-eight hours post-transfection, the cells were treated with E_2_ for 24 h or PPT for 16 h, or DMSO for 24 h. Concurrently, siRNA-A (Santa Cruz) was used as a negative control to demonstrate that there was no effect on expression due to transfection. Following exposure, whole cell extracts were harvested for Western analysis. Figure S3 demonstrates no change in protein expression for ERα. Additionally, the siERα does not show any changes in ERβ expression by Western analysis.

### 4.6. Data Analysis

All experiments were repeated three to six times. Two-tailed Student’s *t* tests were used for comparison between experimental and control groups. A value of *p* ≤ 0.05 was set as the criterion for statistical significance. Bar graphs represented the mean ± SD for three independent experiments. All statistical analysis was performed using SAS 9.1.

## Supplementary Materials

Supplementary materials can be found at http://www.mdpi.com/1422-0067/17/2/158/s1.

## Abbreviations

BRMS1: breast cancer metastasis suppressor 1
CRE: cyclic AMP-response element
DPN: 2,3-bis(4-hydroxyphenyl)-proprionitrile
E_2_: estradiol
ER: estrogen receptor
ERE: estrogen response element
G-1: (±)-1-[(3aR*,4S*,9bS*)-4-(6-Bromo-1,3-benzodioxol-5-yl)-3a,4,5,9b-tetrahydro-3H-cyclopenta[c]quinolin-8-yl]-ethanone
HDAC: histone deacetylase
Hsp: heat shock protein
mSin3: mammalian suppressor of defective silencing 3
PPT: (4,4′,4″-(4-propyl-[1H]-pyrazole-1,3,5-triyl) tris-phenol
