# Supplementary material for: ERα Mediates Estrogen-Induced Expression of the Breast Cancer Metastasis Suppressor Gene BRMS1
Source: Int J Mol Sci. 2016 Jan 26;17(2):158. doi: 10.3390/ijms17020158 (PMC4783892; doi:10.3390/ijms17020158)
Supplement: Supplementary file 1 [file ijms-17-00158-s001.pdf]

Supplementary Materials: ERα Mediates Estrogen-Induced Expression of the Breast Cancer Metastasis Suppressor Gene BRMS1

Hongtao Ma and Lauren S. Gollahon

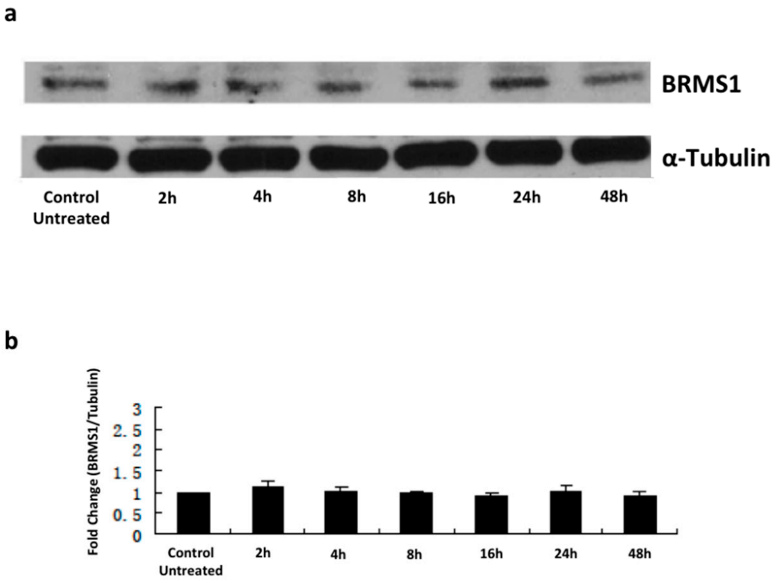

Figure S1. Western analysis of effects of DMSO treatment on BRMS1 protein expression in MCF-7 cells. (**a**) Cells were untreated or incubated with culture media containing 10 nM DMSO for 2, 4, 8, 16, 24, and 48 h. At each time interval, cells were harvested and 20 μg of the total protein was loaded for each sample to determine BRMS1 expression by Western blot. α-tubulin was used as the loading control. Each band present was quantified using Image J and a set area encompassing the BRMS1 band was divided by the same area for α-tubulin from the same lane to determine the relative amount of BRMS1 expression. The control untreated cell extract result value was set to 1 for comparisons; (**b**) The bar graph summarizes BRMS1 protein normalized to α-tubulin from the same lane in three independent experiments. Error bars were the average of three independent experiments ± SD. No significant difference was observed from control. Therefore DMSO controls were treated for 2 hours for subsequent experiments.

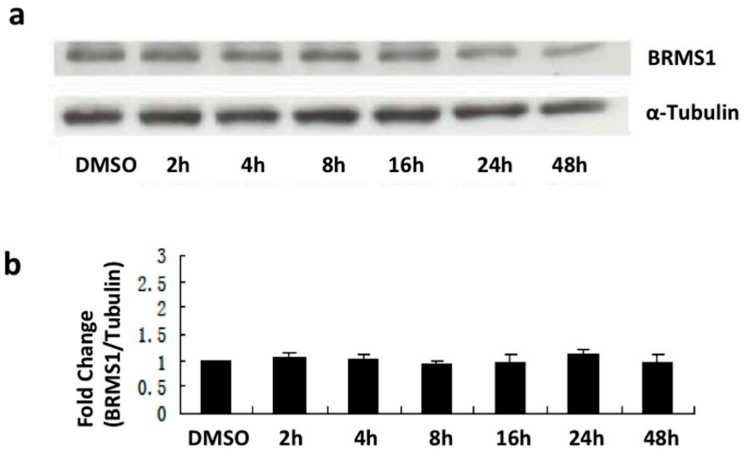

Figure S2. *Cont.*

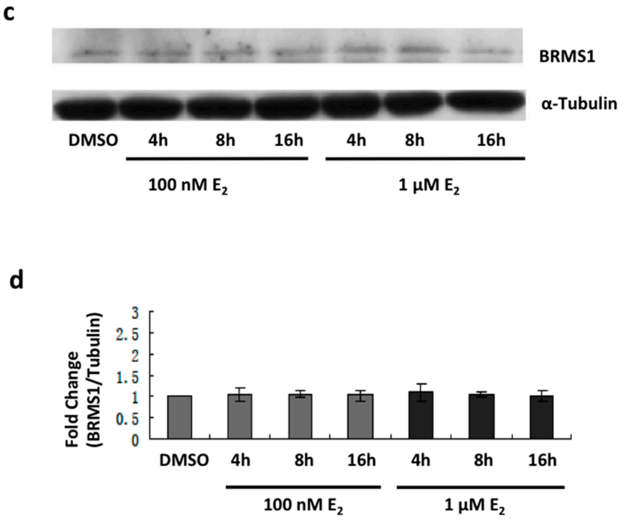

Figure S2. Western blot analysis of BRMS1 protein expression induced by different concentrations of E_2 in TTU-1 cells. (**a**) TTU-1 cells incubated with culture media containing 10 nM E_2 for 2, 4, 8, 16, 24, and 48 h; (**b**) Bar graph summarizes BRMS1 protein normalized to α -tubulin from the same lane from three independent experiments from conditions described in (**a**); (**c**) TTU-1 cells incubated with culture media containing 100 nM E_2 or 1 μM for 4, 8 or 16 h. Control cells were incubated with culture media containing 10 nM DMSO for 2 h. samples were quantified as previously described; (**d**) Bar graph summarizes BRMS1 protein normalized to α -tubulin from the same lane from three independent experiments from conditions described in (**c**). Error bars represent the average of three independent experiments ± SD. No significant difference was observed from control.

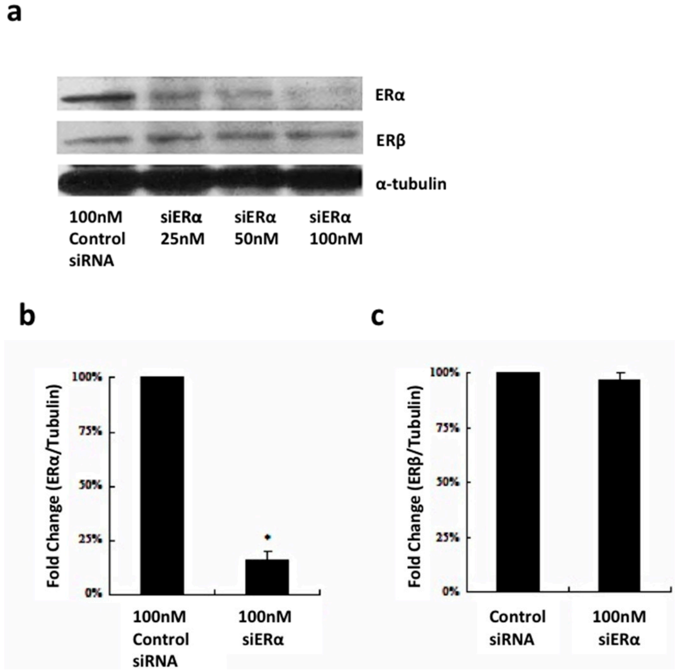

Figure S3. Western blot analysis of ERα and ERβ expression in MCF-7 cells transfected with 25, 50, and 100 nM siRNA against ERα or 100 nM negative control siRNA-A for 48 h. (**a**) Western blot showed protein expression from whole cell extract. α-tubulin was used as the loading contro; The bar graphs summarize ERα (**b**) and ERβ (**c**) protein normalized to α-tubulin from the same lane from three independent experiments. Values with error bars were the average of three independent experiments ± SD. * indicates statistic significance at the $p < 0.05$ compared to DMSO. Each lane was loaded with 40 μg whole cell extract.
